# Supplementary figures and images for: Excessive exercise induces cardiac arrhythmia in a young fibromyalgia mouse model
Source: PLoS One. 2020 Sep 30;15(9):e0239473. doi: 10.1371/journal.pone.0239473 (PMC7526895; doi:10.1371/journal.pone.0239473)

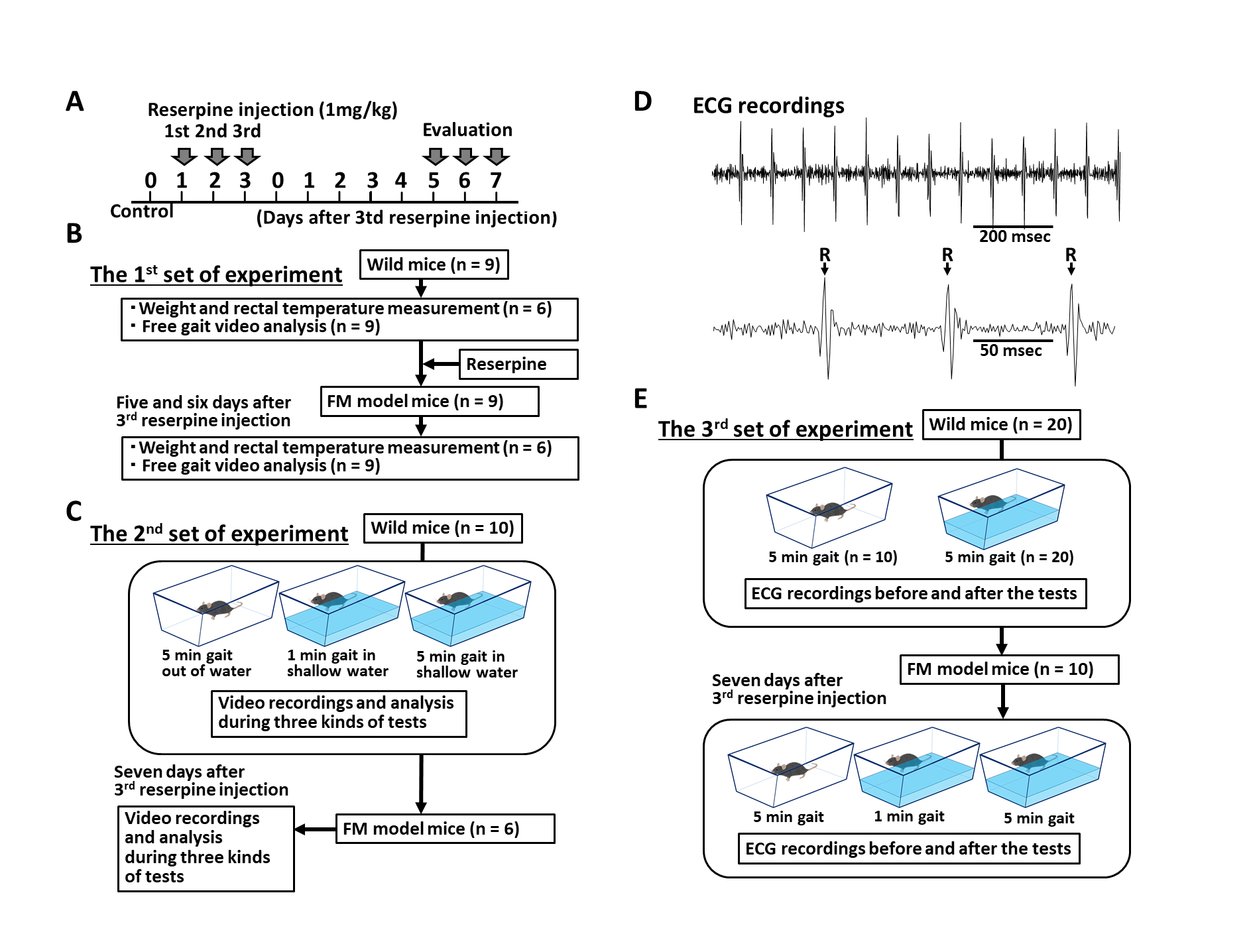

Supplement: S1 Fig — (A) The experimental protocol to produce FM-induced mice. (B) A flow chart of the first experiment to measure body weight and rectal temperature, and video recordings of free gait inside the cage. (C) A flow chart of the second experiment with video recordings before and after the three types of gait tests. (D) Raw data from ECG recordings taken to evaluate cardiac function. R means a negative peak of wave in the ECG. (E) A flow chart of the third experiment for ECG recordings before and after the three types of gait tests. ECG, electrocardiogram; FM, fibromyalgia. (TIF) [file pone.0239473.s001.tif]
